# Contribution of wastewater irrigated vegetables to the prevalence of soil-transmitted helminth infection among female farmers in Addis Ababa, Ethiopia

**DOI:** 10.1186/s41182-024-00604-5

**Published:** 2024-06-06

**Authors:** Bethlhem Kinfu Gurmassa, Sirak Robele Gari, Ephrem Tefera Solomon, Michaela L. Goodson, Claire L. Walsh, Bitew K. Dessie, Bezatu Mengistie Alemu

**Affiliations:** 1 Water and Health, Ethiopian Institute of Water Resources, Addis Ababa University, Addis Ababa, Ethiopia; 2 College of Agriculture and Environmental Sciences, School of Natural Resources Management and Environmental Sciences, Haramaya University, Haramaya, Ethiopia; 3 College of Health and Medical Sciences, School of Medical Laboratory Sciences, Haramaya University, Harar, Ethiopia; 4 Newcastle University Medicine Malaysia, Iskandar Puteri, Johor Malaysia; 5 School of Engineering, Newcastle University, Newcastle Upon Tyne, Tyne and Wear UK; 6 Water and Land Resource Center, Addis Ababa University, Addis Ababa, Ethiopia

**Keywords:** STH, Vegetable, Irrigation, Female, Wastewater

## Abstract

**Background:**

Untreated or inadequately treated wastewater carrying human feces can host helminth eggs and larvae, contaminating the soil and plants that are irrigated with it. In Addis Ababa, farmers use untreated wastewater to grow vegetables; however, there are little data currently available published on vegetables' contribution to the prevalence of helminth among female farmers along the Akaki River, in Addis Ababa, Ethiopia.

**Methods:**

A cross-sectional study was conducted in Addis Ababa City in February 2022. A stratified random sampling method was used to sample farming households. The sample size for each district was determined by a proportional allocation to the total number of households in the area. Two hundred and fifty-two composite vegetable samples and 101 farmers’ stool samples were collected and analyzed for helminth prevalence. Data on socio-demographics were collected by trained data collators using a structured questionnaire. Kato-Katz concentration was used to detect STH from a stool sample. Stata version 14.0 was used to process the data. Poisson regression was used to identify the association between STH prevalence in the vegetable and the farm's stool.

**Results:**

Helminths were found in 67.5% of vegetables sampled and 20.8% of female farmers' stools. *Ascaris lumbricoides* eggs (vegetable 48.4% and stool 9.9%) were identified in all analyzed samples. Hookworm eggs (vegetable 13.1% and stool 8.9%) and *Trichuris trichiura* eggs (vegetable 5.9% and stool 2%) were also isolated. The total number of helminth eggs present in wastewater-irrigated vegetables and female farmers’ stool had a positive association (*p* < 0.05) with a regression coefficient of 1.92 (95%* CI* = 1.56–2.28).

**Conclusions:**

The study found a significant prevalence of helminth infections, particularly *Ascaris lumbricoides*, in stool and vegetable samples irrigated with wastewater. A clear association was found between vegetable production and a higher prevalence of helminth infections among female farmers. Therefore, it is important to ensure that farmers are educated in the importance of food washing and sanitation/hygiene practices when using wastewater irrigation for vegetable crops.

**Graphical Abstract:**

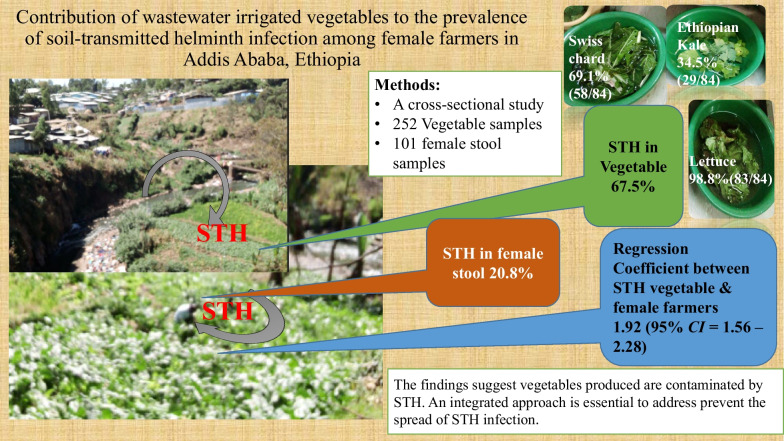

## Highlights

- A cross-sectional research design was carried to investigate the prevalence of helminths in vegetable and stool samples of female wastewater-irrigated vegetable growers.
- STH was found in 67.5% of the vegetables and 20.8% of the stools of the female farmers.
- *Ascaris lumbricoides.* most prevalent in vegetable and stool samples.
- Positive correlation identified between the presence of helminths in vegetables and the level of infection female farmers.
- Risk reduction measures implemented to enforce local authority to enhance adaptive management.

## Background

Wastewater irrigation is used worldwide, with approximately 20 million hectares of agricultural land irrigated by wastewater [1]. Due to limited freshwater resources, countries such as Israel (85%), Saudi Arabia (10%), China (55%), Mexico (60%), and Egypt (59.7%) rely extensively on treated wastewater for irrigation [2]. Irrigation is important in agricultural output in East Africa, contributing to food security and economic development [3]. Several nations in the region, notably Ethiopia, Kenya, Tanzania, Uganda, and Sudan, have put irrigation systems in place to supplement rain-fed agriculture and minimize the effects of climate change [4]. According to a study, using wastewater for irrigation increases soil-transmitted helminth levels in soil and on plants, which can then be transmitted to humans by ingestion of contaminated plants [5]. Female farmers are more vulnerable due to their higher exposure to wastewater during agricultural activities [6].

According to the World Health Organization, soil-transmitted helminth infections affect around 1.5 billion people globally, with the largest illness burden occurring in Sub-Saharan Africa, Southeast Asia, and Latin America [7]. The most common STH species are *Ascaris lumbricoides*, hookworms (*Ancylostoma duodenale* and *Necator americanus*), and *Trichuris trichiura* [8]. The infections are a major contributor to the global illness burden in these regions and can directly or indirectly cause malnutrition and conditions such as anemia in adults or delayed cognitive development in children [8]. In Africa, infection prevalence is especially higher among vegetable growers that use wastewater for irrigation, with studies indicating prevalence rates ranging from 10 to 80% [9].

In Ethiopia, STH infections are a major public health concern, with research revealing high prevalence rates among diverse population groups. A study conducted in Jimma town reported a prevalence of 18.1% helminth infections among peri-urban households [10], in Bahir Dar City 60.6% prevalence of vegetables collected from the local market [11], and in Bench Maji Zone 36% [12] prevalence reported among the community. However, there is a paucity of data on the prevalence of soil-transmitted helminth infections among vegetable farmers who utilize the Akaki River for irrigation purposes in Addis Ababa, Ethiopia's capital city. The River was once a source of life for many communities, but factors like population growth and urban expansion have led to its severe contamination [13]. Studies have identified that the river has lost its natural characteristics and now primarily functions as a wastewater discharge line flowing through the city [13, 14].

Understanding the contribution of wastewater-irrigated vegetables to the prevalence of soil-transmitted helminth among female farmers in Addis Ababa is crucial for designing effective risk mitigation strategies. In this context, the purpose of the study is to investigate the prevalence of helminths in vegetables and female farmers, as well as the epidemiological link between vegetables and the risk of helminth infection among female farmers.

## Methods

### Description of the study area

The study was conducted in Addis Ababa, the capital city of Ethiopia. In 2024, Addis Ababa's metropolitan area population was reported as 5,704,000, a 4.45% increase from 2023 [15]. The city includes well-designed and formally constructed modern neighborhoods in addition to areas that are in less developed, lacking modern ultilities and infrastructure. The city has several water sources, but two main sources are the Little and Great Akaki Rivers, which unfortunately are extremely polluted [13]. The rivers run 76 km and part of this journey is through the city centre, where industrial, domestic, municipal, livestock, and agricultural waste is directly deposited in the river [16]. The river is no longer safe to use for recreational or domestic water use. However, this polluted river water used to cultivate vegetable crops a long the river banks. Lettuce, Ethiopian Kale, Swiss chard, carrot, potato, cabbage, and spinach are the vegetable crops cultivated alonge the river [17].

**Fig. 1:**
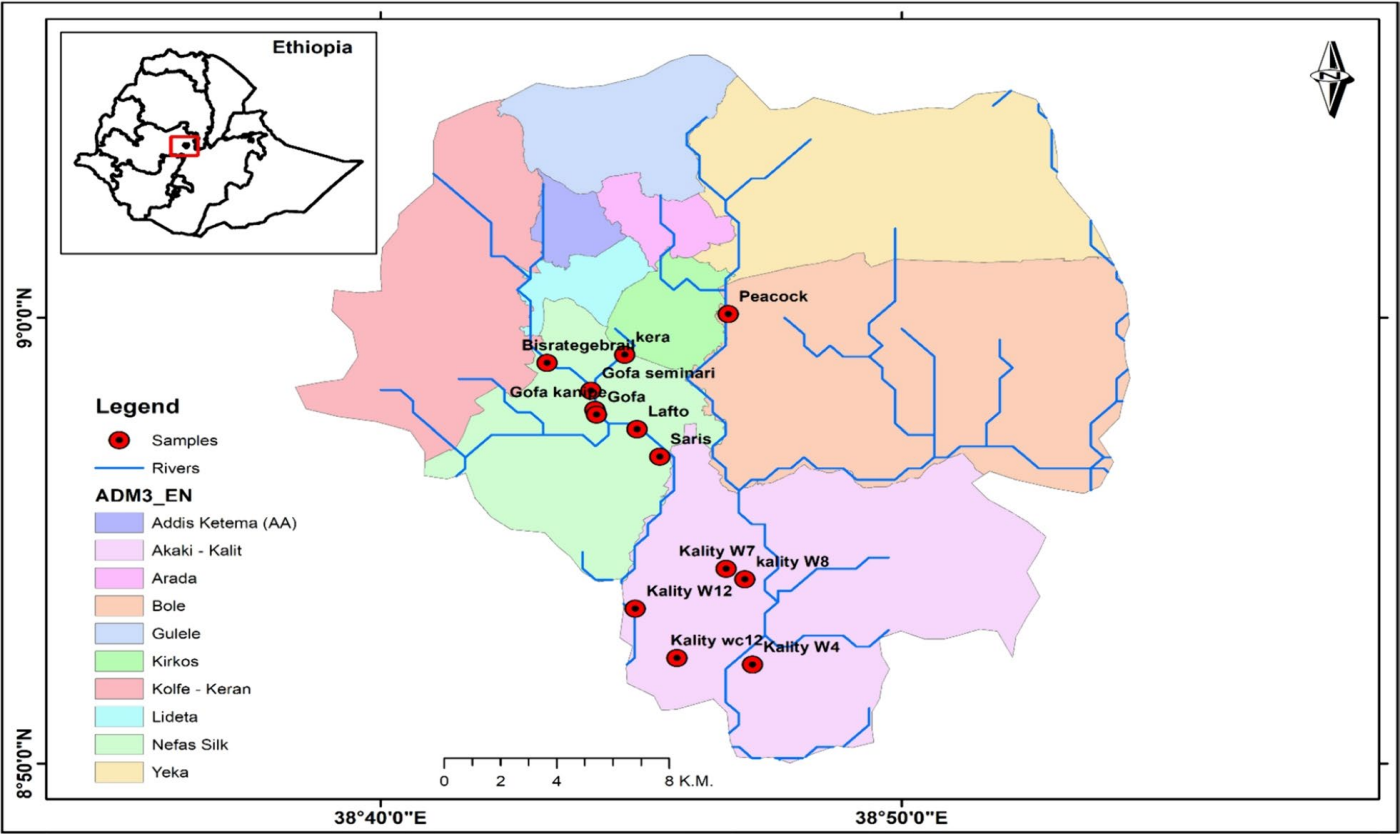
Map of the study area and the sampling sites (Source [18])

The current study covered prominent vegetable production sites, known locally as Bisrategebrail, Gofa, Lafto, Saries, and Kera. They are all irrigated by the Little Akaki River; Peacock-Urael, and Akaki irrigated by the Great Akaki River (Fig. 1). They are located in three sub-cities: Nifas Silk Lafto, Bole, and Akaki Kality.

### Study design and sample period

A cross-sectional study was conducted from November 2021 to February 2022 and investigated the association between helminth infection in female farmers with vegetable produced.

### Source population

The source population considered vegetable farming households located along the Akaki riverbank in Addis Ababa, Ethiopia.

### Study population

The study population considered female vegetable farming community along the Akaki riverbank in Addis Ababa, Ethiopia.

### Inclusion criteria

The following inclusion criteria were considered: (a) located in one of the seven urban vegetable farming districts; (b) being a female farmer who has been cultivating for over years and use Akaki river.

### Exclusion criteria

The following exclusion criteria were considered: (a) farmers who cultivate their land using day laborers and (b) farmers who use both alternate water sources and Akaki river.

### Sample size

The sample size for this study was calculated using the single population formula *n* = *Z*^2^*P*(1-*P*)/*D*^2^. By considering a prevalence for helminth infection 8.02% in a study conducted by Gaidhane et al. in 2022, [19] a marginal error of 5%, a 95% confidence level, the sample size was calculated to be a minimum of 113. Where *Z* = 95% confidence interval, *P* = Estimated prevalence rate, *D* = Marginal of sampling error, *N* = Total study population.

### Sampling technique

A stratified random sampling method was used to sample farming homes, and the sample size for each district was set by a proportionate allocation to the total number of households in the area. To investigate the prevalence of helminths in vegetable samples, 252 composite samples were obtained from a wastewater-irrigated farm using a random sampling technique. The samples were examined in the Ethiopian Public Health Institutes (EPHI) laboratory.

### Data collection

The field farm baseline study employed interviews and observations. Before the survey, a pre-test was done on 5% of the calculated sample size in a nearby farming area that was not included in the study. To determine the prevalence of helminths, a health diary, and a self-reporting sheet were utilized.

### Vegetable sample collection

A composite sample of three vegetables, Ethiopian Kale (Gomen), Lettuce(Selatsa), and Swiss chard (Qosita) was collected using a random sampling technique between 8:00 and 10:00 a.m. To ensure a representative sample, 12 sampling points were selected at each farm site. From each sampling point by going horizontally and vertically twelve composite samples of each type of vegetable were randomly selected. This makes a total of 36 samples per sample point. Old, dead plant material as well as tissue that had been damaged by insects or mechanical equipment were not sampled. Each vegetable sample was placed in a separate sterile polyethylene bag, labeled with a unique number and collection date, and then transported in an ice box to the EPHI laboratory for analysis.

### Parasite detection

A sample of 250 g of each vegetable was weighed in the lab then, rinsed completely in a saline solution (0.85% NaCl) and, soaked in plastic containers. The vegetable soil-derived fragments and debris from the washing saline solution were left to settle overnight [20].

A modified Bailenger method was used for centrifugation [20]. Following overnight settling, the supernatant was removed from the samples, leaving sediment at the base of the containers. The sediment was then transferred to three 50 ml centrifuge tubes and centrifuged at 1500 RPM for 3 min. Then, a 15 ml acid/alcohol buffer solution and around 5 ml ethyl acetate were added to the sediment and agitated, allowing gaseous reaction products to escape intermittently. The mixture was then centrifuged at 2200 RPM for 3 min. Finally, the diphasic supernatant was removed, leaving around 1 ml of sediment or suspension for microscopic investigation. Helminth eggs were identified in a light microscope with a magnification of 10× and 40× objective lenses. Helminth eggs were then identified, enumerated, and quantified based on shape and size.

### Stool sample collection and examination

Each participant provided a 2 g stool sample, collected using a clean, leak-proof, screw-cap stool cup labeled with a unique identifier. Specimens were then labeled and delivered to the laboratory. According to WHO recommendations, a double Kato-Katz smear was performed for each sample and inspected microscopically to detect helminth eggs in the stool sample [20]. The Kato-Katz slides were produced as soon as the stool samples arrived in the laboratory.

### Data quality management

To verify for data collection errors, data were entered into the statistical software EpiData version 3.1. Errors were corrected by revisiting the original questionnaire and responses. For laboratory data, a laboratory data collection sheet was employed, and the data were promptly entered into Excel. The laboratory equipment used was calibrated.

### Data analysis

Data were checked for completeness and consistency. Cleaned data were entered into EpiData Version 3:1 and exported to STATA Version 14.0 for analysis. To describe the presence of helminths in stool and vegetable samples, descriptive data analysis was utilized. To describe the presence of helminths in stool and vegetable samples, descriptive data analysis was utilized. In addition, the relationship between helminth loads in vegetable samples and helminth per gram of stool matter from female farmers was evaluated using Poisson regression analysis. Statistical significance was set at *p* < 0.05.

## Results

### The socio-demographic characteristics of the study participants

Study participants were aged between 31 and 40 (52.5%) of which, 64.4% were illiterate. Sixty points four percence of the participants had a family size of four to six individuals, lived in 1–2 room houses, and the largest group earned between 1.80$ and 5.39$ (35.6%). In this study, 39.6% of participants were married and 32.7% were widowed (Table 1).

**Table 1:** Socio-demographic characteristics of the female farming community in Addis Ababa, from November 2021 to February 2022, Addis Ababa, Ethiopia

Characteristics		Frequency (%)
Age	< 30	5 (4.9)
	31–40	53 (52.5)
	41–50	33 (32.7)
	> 50	10 (9.9)
Education	Primary education	25(24.7)
	Secondary & above	11 (10.9)
	Illiterate	65 (64.4)
Family size	1–3	19 (18.8)
	4–6	61 (60.4)
	> 6	21 (20.8)
Number of room	1–2	45 (44.5)
	3–4	41 (40.6)
	> 4	15 (14.9)
Income (ETB)	< 1000	31 (30.7)
	1001–3000	36 (35.6)
	3001–5000	19 (18.8)
	> 5000	15 (14.9)
Marital states	Single	9 (8.9)
	Married	40 (39.6)
	Divorced	19 (18.8)
	Widowed	33 (32.7)

### Prevalence of helminths in vegetable samples

A total of 252 wastewater-irrigated vegetable samples were tested for the presence of helminths. In total, the overall prevalence of helminths identified in our study sample was 67.5% (170/252). All three types of vegetables were contaminated with at least one of the three soil transmitted helminths. *Ascaris lumbricoides* were found to have the highest prevalence rate (48.4%), followed by Hookworm (13.1%) then *Trichuris trichiura* (5.9%) (Table 2). Lettuce showed the highest rate of prevalence for all three helminths, with 98.8% of samples found to be positive for at least one STH. Swiss chard had the second-highest prevalence of helminths, with 69.1% of samples testing positive, and Ethiopian kale had the lowest prevalence, with 34.5% of samples testing positive (Table 2).

**Table 2:** Prevalence of parasites in wastewater irrigated vegetables in Addis Ababa, Ethiopia, from November 2021 to February 2022

Vegetables	Number of samples examined	*Ascaris lumbricoides *(%)	Hookworm (%)	*Trichris trichiura* (%)	Total (%)
Lettuce	84	61 (72.6)	15 (17.9)	7 (8.3)	83 (98.8)
Swiss chard	84	43 (51.2)	11 (13.1)	4 (4.8)	58 (69.1)
Ethiopian Kale	84	18 (21.4)	7 (8.3)	4 (4.8)	29 (34.5)
Prevalence	252	122 (48.4)	33 (13.1)	15 (5.9)	170 (67.5)

As shown in Table 3, the highest prevalence of *Ascaris lumbricoides* was found in Bisrategebrail, Kera, and Akaki, with 66.7%, 63.8%, and 63.8% of vegetables testing positive, respectively. For Hookworm, Peacock Urael and Akaki had the highest prevalence, with 13.8% and 16.6% of the vegetables testing positive, respectively, Gofa had the lowest prevalence at 5.5% (Table 3).

**Table 3:** Prevalence of parasites in vegetable samples from seven wastewater irrigation farming sites in Addis Ababa, Ethiopia, from November 2021 to February 2022

Site	Number of samples examined	*Ascaris lumricoides* (%)	Hookworm (%)	*Trichris trichiura* (%)	Total (%)
Bisrategebrail	36	24 (66.7)	7 (19.4)	4 (11.1)	35 (97.2)
Gofa	36	10 (27.8)	2 (5.5)	1 (2.7)	13 (36.1)
Lafto	36	13 (36.1)	6 (16.6)	1 (2.7)	20 (97.2)
Saris	36	11 (30.5)	4 (11.1)	3 (8.3)	18 (50)
Kera	36	23 (63.8)	3 (8.3)	1 (2.7)	27 (75)
Peacock Urael	36	18 (52)	13 (13.8)	2 (5.5)	25 (69.4)
Akaki	36	23 (63.8)	16 (16.6)	3 (8.3)	32 (88.8)

### Prevalence of helminths in the farmer’s stool

**Fig. 2:**
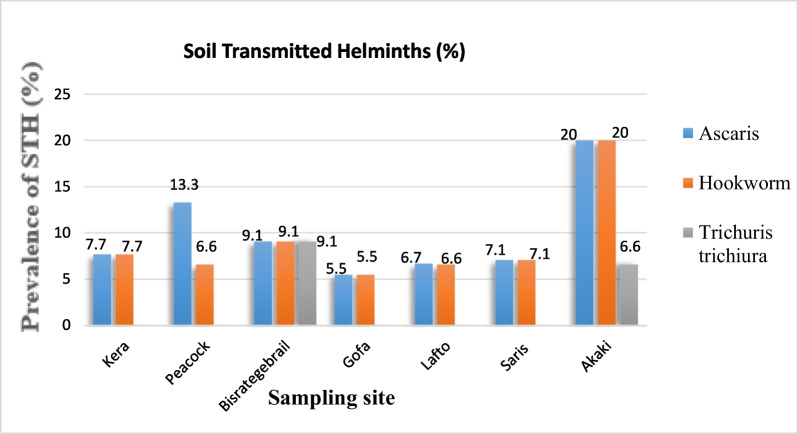
Prevalence of helminths in female farmers’ stools from seven farming sites in Addis Ababa from November 2021 to February 2022

The prevalence of soil transmitted helminths in the study sample was 20.8%, with 21 cases out of 101 samples testing positive. The most common helminth infection was *Ascaris lumbricoides*, which was found in 10 female farmers (9.9%), followed by hookworm in 9 farmers (8.9%), and *Trichuris trichiura* in 2 farmers (2%) (Fig. 2).

One female farmer in Bistrategebrale (9.1%) tested positive for all three parasites. Figure 2 shows that the Akaki site has the highest prevalence of *Ascaris lumbricoides* (20%), hookworm (20%), and *Trichuris trichiura* (6.6%). The prevalence of *Ascaris lumbricoides* was found to be higher in Peacock and Bisrategebrail than in other sites.

### Association between helminth eggs in wastewater irrigated vegetables, and female farmers’ stool

The number of helminth eggs in wastewater-irrigated vegetables and female farmers’ stools showed a statistically significant positive relationship (*p* < 0.05), with a regression coefficient of 1.92 (95%* CI* = 1.56–2.28) (Table 4). When comparing lettuce to other vegetables, the sample exposure resulted in a 1.88 (95%* CI* = 1.67–2.23) Poisson regression coefficient (Table 4).

**Table 4:** Association between helminth eggs in wastewater-irrigated vegetables and female farmers’ stools in Addis Ababa from November 2021 to February 2022

STH in vegetable samples versus female farmers’ stool	Coefficient	95% Confidence Interval	*p-*value
Total vegetables	1.92	1.56–2.28	0.001
Lettuce	1.88	1.67–2.23	0.005
Swiss	1.55	1.28–1.82	0.018
Ethiopian kale	1.22	0.77–1.67	0.024

## Discussion

Vegetable consumption is highly beneficial for health maintenance and disease prevention. On the other hand, they may serve as a source of infection for a variety of parasite infections. Detecting parasites in vegetables is critical for parasitic illness prevention and control [21]. In this study prevalence of STH was found on the harvested leafy vegetables. Due to their larger surface area and morphological ability to trap soil and sediment; this creates favorable conditions for STH eggs to adhere to the surface of the vegetables [5]. Moreover, these vegetables are often grown in loose soil, which is more conducive to the survival and growth of STH eggs [22]. In this study site, irrigation methods used during the seedling of leafy vegetables are often overhead. The contaminated water comes into contact with the leaves and remains there. This increases the risk of STH contamination in leafy vegetables [23].

The prevalence of helminth detected in the vegetable sample was 67.5% which is comparable with previous findings of Desta et al*.,* 61% [17], and a study conducted in Dessea, Ethiopia 63.4% [12]. However this result is higher than studies conducted in different parties of Ethiopia; in Bardar, the prevalence of STH was 39.1% [11], Dire Dawa, 47.3% [24], Arba Minch 54.4% [25]. The difference could be due to variations in sanitation and hygiene practices, geographical locations, climatic and environmental conditions, methods used for the detection of STH parasites, and socioeconomic status.

This study provided data on the prevalence of helminth infection among urban female vegetable farmers of Addis Ababa city. *Ascaris lumbricoides* and hookworm were the most prevalent infection in the population studied, consistent with global trends. Eight hundred and 600 million people were infected with *Ascaris lumbricoides* and hookworm at the global level respectively [26]. The low-frequency *Trichuris trichiura* observed might be due to their minimal dispersion as a single female worm of *Trichuris* liberates relatively fewer numbers eggs (20,000 eggs per day) [26].

The current study found a helminth prevalence of 20.8%, among female farmers growing vegetables using wastewater as an irrigation source. This figure is similar to a study conducted in Babile, Eastern Ethiopia which reported a prevalence of 20.8% [27] and comparable with the study in rural Vietnam 19.2% [28]. According to studies female farmers in general more vulnerable to soil-transmitted helminth infections due to their frequent contact with contaminated soil through agricultural activities, such as planting, weeding, and harvesting [29, 30]. They have less access to sanitation facilities and hygiene education compared to their male counterparts, which may increase their risk of STH infections. In addition, female farmers may be responsible for caring for children and other family members, which may limit their time and resources to practice good personal hygiene [6].

In the existing study, a statistically significant (*p* < 0.05) positive regression coefficient suggests that there is a positive association between STH prevalence in the three vegetable and female vegetable farmers. The coefficient indicates that an increase in STH prevalence in the vegetable sample is associated with an increase in the number of cases of STH infection among female farmers. The positive association is due to poor sanitation practices in the surrounding areas. The absence of proper sanitation facilities for female farmers and their families, combined with their defecation practices in nearby bushes, has led to contamination of the irrigation water and soil. The contaminated water and soil have then contributed to the higher number of helminths found in vegetables. The confirmation of a similar increment in parasite prevalence in the surrounding areas through stool surveys further supports that poor sanitation practices are contributing to the contamination of the vegetables.

Vegetables are eaten raw or lightly cooked to preserve taste and this practice may favor the transmission of STH infections. lettuce is eaten raw, which is the most frequently contaminated sample in the study sites. The leaves of lettuce have a favorable structure for the retention of soil particles, as it has numerous crevices and folds, which can provide shelter protection to helminth eggs [17]. Besides, helminths infect vegetables in a variety of ways, including growth, harvesting, handling, or distribution, which poses a significant occupational health risk.

This study has the major benefit of clearly demonstrating the link between helminth infections in female farmers and the use of wastewater for vegetable irrigation. The detailed collection of vegetable samples along the river line provides valuable information for environmental and community health surveillance.

### Limitations of the study

The study's limitations, such as its focus on only leafy vegetables, farmers, and the absence of helminth infection identification in the vegetable market sellers, may impact the generalizability of the findings. The analysis only captures the relationship between wastewater-cultivated vegetable versus STH infection among female farmers, other factors may also influence the prevalence of STH infection. Therefore, further robust evidence-providing research is needed to better understand the complex relationships between STH in vegetables and the prevalence of STH infection among female farmers.

## Conclusion

The study found a high prevalence of helminths in wastewater-irrigated vegetables. *Ascaris lumbricoides* was found to be the most prevalent helminth, followed by hookworm and *Trichuris trichiura*. The prevalence of helminth infections varied between study sites, with the highest prevalence of *Ascaris lumbricoides,* hookworm, and *Trichuris trichiura* being observed in Akaki. Lettuce was the most frequently contaminated sample in the study sites. The study found a statistically significant positive relationship between vegetable production and female farmers’ risk of infection. The farmers had a higher chance of developing STH infections by working on the farm.

The findings of this study suggest the government deworming program must include agricultural workers around the Akaki. Furthermore, comprehensive health education should be implemented among the farming community and people living in the surrounding to raise awareness of the risks of working and living in a helminth-contaminated environment. Improved hygiene practices during vegetable production and processing are also needed to reduce the risk of prevalence and ensure the safety of the food supply chain. More effective waste treatment and management by the city sanitation office would also reduce helminth transmission risk.

Further research may be necessary to understand alternative risk factors associated with helminth infections among farmers and the potential effects of helminth infections further down the food supply chain. It is vital to study the utilization of low-cost and scientifically acceptable wastewater treatment technologies (such as constructed wetlands and phytoremediation) and consider restricting the use of wastewater irrigation for vegetables that are eaten raw or uncooked, particularly in the rainy season where there is a high seasonal variation of helminth prevalence.

### Operational definitions

Farmers: those female communities cultivate vegetables along the Akaki Riverside.

Wastewater: the untreated Little and Grate Akaki River.

Permanent: farmers have lived along the Akaki Riverside for more than 10 years.

## Data Availability

The data used in this manuscript are not publicly available due to ongoing analyses. Data presented in this study are available from the corresponding author upon reasonable request.

## Abbreviations

STH: Soil-transmitted helminths
WHO: World Health Organization
EPHI: Ethiopian Public Health Institute
FDRE: Federal Democratic Republic of Ethiopia
UNDP: United Nations Development Programme
